# Distribution Locational Marginal Pricing under Generation and Network Scarcity Conditions

**DOI:** 10.1007/s40518-025-00282-9

**Published:** 2025-12-16

**Authors:** Zejun Ruan, Anthony Papavasiliou, Mehdi Madani

**Affiliations:** 1 Electrical and Computer Engineering, National Technical University of Athens, 9 Iroon Polytechniou St, Athens, 15772 Greece; 2 N-Side, Louvain-la-Neuve, Brussels, 1348 Belgium

**Keywords:** Reserve deliverability, TSO-DSO coordination, price propagation, scarcity pricing, co-optimization of energy and reserve

## Abstract

**Purpose of review:**

This paper addresses the integration of reserve scarcity pricing into distribution locational marginal prices (DLMPs) by proposing a computationally tractable formulation of reserve deliverability. The primary goal is to understand how network congestion and generation scarcity affect DLMPs and to evaluate the effectiveness of different market design approaches in providing accurate investment signals in distributed energy systems.

**Recent findings:**

Recent research has introduced flexibility platforms and models that attempt to integrate distributed energy resources within market operations. This work builds upon the Caramanis model, and introduces an inscribed-boxes formulation that allows for scalable application to meshed networks. The proposed model is shown to be equivalent to existing approaches on radial networks and offers computational tractability. Furthermore, it enables detailed analysis of DLMP pricing patterns under congestion and various energy and reserve flow scenarios.

**Summary:**

The analysis reveals that accounting for reserve deliverability significantly impacts DLMPs and investment incentives. The findings emphasize that without incorporating network constraints and scarcity pricing, investment signals may be distorted, potentially leading to suboptimal infrastructure placement.

## Introduction

### Motivation

In recent years, global power systems are rapidly transitioning towards the integration of Distributed Energy Resources (DERs), due to policy directives [1] and technological advances. However, within distribution networks, the substantial penetration of DERs can lead to technical complexities and operational challenges. In a forward-looknig scenario where a significant amount of flexibility is sourced from resources connected at distribution systems, it is plausible that power flows resulting from the activation of such flexible resources can cause network congestion and voltage violations. In response to such challenges, Europe has put forward a solution advocating for DSOs to have an active role in managing DERs services, while also integrating network constraints [2]. The inherent influence of DSOs on the provision of services by DERs mandates a fundamental re-evaluation of transmission and distribution interaction. Therefore, effective TSO-DSO coordination is vital for both realizing the DSO’s function and ensuring the operational efficiency of power systems [3].

In Europe, a prominent strategy for mobilizing distribution system flexibility involves the implementation of flexibility platforms. Their rapid growth is attested by the emergence of a number of commercial platforms. A notable but non-exhaustive list of platforms that have been or are at least partially active in Europe is presented here: (i) ENERA, a pilot platform in Germany implemented by the EPEX which has been discontinued [4]; (ii) a number of platforms implemented by software vendor Picloflex in the UK, the US, Australia, Portugal, and Italy [4, 5]; (iii) NODES, which was formerly owned by NordPool , is currently owned by independent entity Nodes, and is currently deployed in Norway, Sweden and Canada [4–7]; (iv) EDGE, a pilot owned by Italian DSO E-Distribuzione and is active in three provinces of Italy [8]; (v) RomeFlex, which is a joint venture between Italian DSO Areti and the Italian power exchange GME and is deployed in the region of Rome [9]; (vi) GOPACS, which is owned jointly by Dutch TSO Tennet and five Dutch DSOs [4, 5]; (vii) the flexibility platform of the French DSO Enedis, which is active throughout France [10]; (viii) the local energy market of the European power exchange, which is active in the UK [11].

The aforementioned flexibility platforms vary in terms of market organization. There is a wide diversity of design choices that have been made. Indicative points of differentiation include: (i) the provided services (which commonly include distribution congestion management, but also restoration and reactive power provision); (ii) the targeted horizon (some platforms offer long-term reservations, others offer short-term activation); (iii) the degree of integration to existing wholesale markets (ranging from none to tight-knit integration with intraday markets); (iv) the criteria for matching flexibility service providers to network operators (ranging from auctions that are based on purely economic criteria to a mix of technical and economic evaluations of long-term offers); (v) the market organization (ranging from continuous matching to closed-gate auctions).

In this paper we aim to contribute to the debate on market design for flexibility platforms. Despite their diversity, all mentioned platforms share two important common features: there is an intrinsic locational component (resources need to indicate not only what they can offer, but also where they are located), and there is a critical dependence on performing when needed (with exclusion clauses foreseen for resources that systematically fail to deliver on their promised flexibility). We consider the location and pay-for-performance attributes as being crucial, not only for ensuring that the resources can deliver reliably on the short term, but also so that the design of these flexibility markets can be set up in a way that provides signals for investing in the appropriate degree of flexibility at the appropriate locations of the network. Our proposed formulation for TSO-DSO integration presented in this paper addresses these attributes through the resolution of an integrated optimal power flow, which comprehensively accounts for both transmission and distribution networks. The proposed marke clearing model includes an operating reserve demand curve (ORDC) within the high-voltage transmission system [12] and facilitates endogenous reserve deliverability. This ultimately enables the formation of distribution locational marginal prices and ensures that reserve scarcity adders are propagated exclusively to network segments where flexibility resources can indeed provide useful services to the grid.

### Practical Applications

The representation of reserve deliverability through inscribed boxes and the proposed hierarchical coordination framework between TSOs and DSOs have emerged from applied collaborative research [13–17]. The inscribed boxes methodology for ensuring network-feasible reserve activation was developed in conjunction with the SDAC expert group, focusing on meshed transmission networks, and has subsequently been incorporated into regulatory analyses [13–15]. In parallel with the above assignments, in the context of collaboration with the Norwegian TSO Statnett and the DSO Tensio [16], our team further developed [17] the idea of a hierarchical TSO–DSO coordination mechanism detailed in this paper. During this work, it became evident that the previously developed "inscribed boxes" concept could be directly applied within TSO-DSO coordination models to integrate reserve deliverability at the distribution level. Subsequently, this realization formed the connection to the framework proposed by Hogan and Caramanis [18]. Furthermore, a subsequent collaboration with the Greek DSO HEDNO involved a comprehensive survey of EU flexibility platforms. This survey inspired the brief review in Section 1.1, thereby positioning the proposed design against contemporary European practices and clarifying both its advantages and limitations.

### Contributions

In this work we revisit the model of [18] which inspires our proposed market clearing model. In revisiting the model of [18], we uncover an equivalent model that emerges from an entirely different view of reserve deliverability^1^, namely from the point of view of inscribing a box on the polyhedral set of feasible reserve transactions between distribution system flexibility service providers and network operators. Our novel model, first introduced in [20], relies on results from computational geometry [21], and can generalize beyond the model of Caramanis to meshed transmission networks [15, 22]. This generalization goes beyond the capabilities of the model of [18] as it overcomes the exponential growth in the number of power flow scenarios that need to be considered in meshed networks. The inscribed boxes method for meshed networks is discussed in [13] and further in [20]. Nevertheless, we further validate the approach, but also establish an equivalence between our model and that of [18] for radial networks.

With our proposed model at hand, we also revisit the properties of the market clearing prices that emerge from our model. We are able to fully characterize the dual multipliers of the underlying model, and this also allows us to uncover patterns of scarcity price propagation in radial distribution networks.

Finally, we demonstrate the relevance of our model in terms of providing appropriate long-term investment signals by conducting a case study on a realistic model of the Belgian system. We demonstrate how alternative combinations of market design choices (presence or lack of scarcity pricing, presence or lack of reserve deliverability) can affect the financial viability of a flexibility service provider that considers investing at different parts of a distribution network.

## Models

In this section we formulate our proposed model for the coordinated clearing of transmission and distribution while accounting for reserve deliverability. We then motivate the representation of reserve deliverability through an approximation based on inscribed boxes in [20]. We then cast the model of [18] in our notation in Section 2.1. Subsequently, we present our inscribed boxes formulation in Section 2.2. Table 1 provides an overview of the notation utilized within our models.

**Table 1:** Nomenclature that is used in the models

Symbol	Description
*N*	Set of buses in transmission and distribution networks
$\textit{US}_n$	Set of generators connected at bus *n*
*IS*	Transmission buses that absorb reserve flows
*S*	Set of scenarios: 0 (base), 1 (activation)
*K*	Set of lines in the network
*RL*	Segments in operating reserve demand curve
$PR_i$	Reserve price of ORDC segment *i*
$MC_g$	Marginal cost of generator *g*
$QR_i$	Reserve quantity for ORDC segment *i*
$Q^+_n$	Maximum active power a generator at bus *n* can offer
$Q^-_n$	Minimum active power a generator at bus *n* can operate at
$F^+_k$	Max capacity of line *k*
$P_n$, $D_n$	Day-ahead power production/consumption at bus *n*
$B_g$	Bus where generator *g* is located
$FB_k$, $TB_k$	From-bus and to-bus of line *k*
$p_g$	Real-time power production of generator *g*
$P^0_g$	Day-ahead commitment of generator *g*
$I^+_g(s)$	Upward reserve indicator for generator *g* in *s*
$I^-_g(s)$	Downward reserve indicator for generator *g* in *s*
$pr^{up}_{n,s}$	Upward reserve activation at bus *n* and scenario *s*
$pr^{dn}_{n,s}$	Downward reserve activation at bus *n* and scenario *s*
$r^{up}_n$	Upward reserve offered at bus *n*
$r^{dn}_n$	Downward reserve offered at bus *n*
$dr^{up}_l$	Upward reserve demand consumed in order *l*
$dr^{dn}_l$	Downward reserve demand consumed in order *l*
$imb_{l,s}$	Reserve imbalance absorbed in scenario *s*
$ne_n$	Energy injection at bus *n*
$ne_{n,s}$	Energy injection at bus *n*, scenario *s*
$nr^{up}_n$	Upward reserve injection at bus *n*
$nr^{dn}_n$	Downward reserve injection at bus *n*
$f_{k,s}$	Power flow on line *k* in scenario *s*
$fr^+_k$	Reserve flow in reference direction of line *k*
$fr^-_k$	Reserve flow opposite to reference direction of line *k*

### Caramanis Model

We first proceed with formulating the model of [18]. We take the liberty of adapting the model to our own notation, but we preserve the central idea of reserve deliverability that is conceived by [18]. Concretely, reserve scarcity prices at distribution level in the approach of [18] are generated by (i) introducing an ORDC at the high-voltage transmission system and (ii) representing the fact that the commitment of flexible resources in the distribution system can potentially take up space on distribution lines. The latter is captured by considering two possible activation patterns that result in the maximum possible loading of distribution lines. The first worst-case activation pattern ($s = 1$) corresponds to a full upstream stress on distribution lines through a *simultaneous* upward activation of *all* distribution-level flexibility resources, while the second worst-case activation pattern ($s = 2$) corresponds to full downstream stress on distribution lines through a *simultaneous* downward activation of *all* distribution-level flexibility resources. Intermediate levels of activation are guaranteed to be feasible as long as the aforementioned activation patterns are feasible. The model is concretely formulated as follows, and we will refer to it throughout the rest of the paper as the “Caramanis” model:

1 $$\begin{aligned}& \underset{p,r^{dn},r^{up},dr^{up},dr^{dn},pr^{up},pr^{dn},ne,f, imb}{\text {max}}\sum _{l \in RL} {PR_l \cdot dr^{up}_l}\\&+\sum _{l \in RL'} {PR_l \cdot dr^{dn}_l}\nonumber -\sum _{g \in US: B_g = n} {MC_g \cdot p_{g}} \end{aligned}$$

2 $$\begin{aligned}&pr^{up}_{g,0} = 0, \quad g\in US \end{aligned}$$

3 $$\begin{aligned}&pr^{dn}_{g,0} = 0, \quad g\in US \end{aligned}$$

4 $$\begin{aligned}&imb_{l,0} = 0, \quad l\in IS \end{aligned}$$

5 $$\begin{aligned}&pr^{up}_{g,s} = I^+_g(s)\cdot r^{up}_g, ~s \in S, ~g \in US \end{aligned}$$

6 $$\begin{aligned}&pr^{dn}_{g,s} = I^-_g(s)\cdot r^{dn}_g, ~s \in S, ~g \in US \end{aligned}$$

7 $$\begin{aligned}& (\rho _{n,s}):~\sum _{g \in US: B_g = n} (p_{g} + pr^{up}_{g,s} + P^0_g)\\&+ P_n - \sum _{g \in US: B_g= n} pr^{dn}_{g,s} - D_n\nonumber \\&-\sum _{l \in IS: B_l = n} imb_{l,s}= ne_{n,s}, \quad n \in N,~ s \in S \end{aligned}$$

8 $$\begin{aligned} (\psi _k):&~f_{k,s} = \sum _{n \in N} PTDF_{kn} \cdot ne_{n,s},\quad k \in K , ~s \in S \end{aligned}$$

9 $$\begin{aligned} (\sigma _s):&~\sum _{n \in N} ne_{n,s} = 0,\quad s \in S \end{aligned}$$

10 $$\begin{aligned} (\lambda R^{up}):&~\sum _{l \in RL} dr^{up}_l - \sum _{g \in US} r^{up}_g = 0 \end{aligned}$$

11 $$\begin{aligned} (\lambda R^{dn}):&~\sum _{l \in RL'} dr^{dn}_l - \sum _{g \in US} r^{dn}_g = 0 \end{aligned}$$

12 $$\begin{aligned} (\phi _{k,s}^+):&~f_{k,s} \le F^+_k,\quad k \in K, ~s \in S \end{aligned}$$

13 $$\begin{aligned} (\phi _{k,s}^-):&~-f_{k,s} \le F^+_k, \quad k \in K, ~s \in S \end{aligned}$$

14 $$\begin{aligned} (\mu ^{up}_g):&~P^0_g + p_g + r^{up}_g \le Q^+_g, \quad g \in US \end{aligned}$$

15 $$\begin{aligned} (\mu ^{dn}_g):&~P^0_g + p_g - r^{dn}_g \ge Q^-_g, \quad g \in US \end{aligned}$$

16 $$\begin{aligned} (v^{up}_l):&~dr^{up}_l \le QR_l, \quad l \in RL \end{aligned}$$

17 $$\begin{aligned} (v^{dn}_l):&~dr^{dn}_l \le QR_l, \quad l \in RL' \end{aligned}$$

18 $$\begin{aligned}&dr, r \ge 0 \end{aligned}$$

One of the most crucial parts of this model is the scenario-dependent parameter *I*, which is an indicator of activation of reserve capacity for a generator^2^ in a certain scenario. The objective function of the model (1) maximizes welfare. The first two terms are the system operator benefit acquired by fulfilling the demand of an operating reserve demand curve and the latter is the operational cost. Eq. 7 represents the energy balance for each scenario in each node, *pr* is the activation of reserve by a generator in a given scenario and *imb* is a variable that is introduced in order to absorb the activation of reserves in a given scenario. This variable essentially represents the realized consumption of activated reserves at the transmission level. Eqs. 10 and 11 are upward and downward reserve trade balance equations. Eqs. 12 and 13 correspond to line capacity constraints for the flow variable $f_{k,s}$ for each line *k* and scenario *s* and are thus representing network restrictions when reserves are activated. Eqs. 14 and 15 correspond to generator capacity constraints. The remaining (16), (17) represent the maximum reserve demand of the *l*-th order of the ORDC.

### Inscribed-boxes Model

In this section we proceed to present an alternative formulation of a co-optimized energy-reserves transmission-distribution coordination model. It preserves the same idea as [18], but approaches the formulation from an entirely different angle, namely the idea of inscribed boxes that is presented in [20].

19 $$\begin{aligned}& \underset{p,r^{up},r^{dn},dr^{up},dr^{dn},ne,nr^{up},nr^{dn},fr^{+},fr^{-}}{\text {max}} \sum _{l\in RL} PR_l \cdot dr^{up}_l\\&+ \sum _{l\in RL'} PR_l \cdot dr^{dn}_l \nonumber - \sum _{g\in US} MC_g \cdot p_g \end{aligned}$$

20 $$\begin{aligned} (\lambda _n):~&ne_n = \sum _{g\in US : B_g=n} (p_g + P^0_g) + P_n - D_n, \quad n \in N \end{aligned}$$

21 $$\begin{aligned} (\lambda R^{up}_n):~&nr^{up}_n = \sum _{g\in US : B_g=n} r^{up}_g - \sum _{l\in RL : B_l=n} dr^{up}_l , \quad n \in N \end{aligned}$$

22 $$\begin{aligned} (\lambda R^{dn}_n):~&nr^{dn}_n = \sum _{g\in US : B_g=n} r^{dn}_g - \sum _{l\in RL' : B_l=n} dr^{dn}_l, \quad n \in N \end{aligned}$$

23 $$\begin{aligned} (\sigma ):~&\sum _{n\in N} ne_n = 0 \end{aligned}$$

24 $$\begin{aligned} (\kappa ^{up}_n):~nr^{up}_n =&\sum _{k\in K : FB_k=n} fr^{{up}^+}_k - \sum _{k\in K : TB_k=n} fr^{{up}^+}_k \nonumber \\&+\sum _{k\in K : FB_k=n} -fr^{{up}^-}_k - \sum _{k\in K : TB_k=n} -fr^{{up}^-}_k, \quad n \in N \end{aligned}$$

25 $$\begin{aligned}&(\kappa ^{dn}_n):~nr^{dn}_n \\&= -\sum _{k\in K : FB_k=n}fr^{{dn}^+}_k + \sum _{k\in K : TB_k=n} fr^{{dn}^+}_k \nonumber \\&+ \sum _{k\in K : FB_k=n} fr^{{dn}^-}_k - \sum _{k\in K : TB_k=n} fr^{{dn}^-}_k, \quad n \in N \end{aligned}$$

26 $$\begin{aligned}& (\phi ^{{up}^+}_k):~\sum _{n\in N} PTDF_{n,k} \cdot ne_n \\&+ \sum _{k' \in K} \max (A_{k,k'},0) \cdot fr^{{up}^+}_{k'}\nonumber \\&+\sum _{k' \in K} \max (-A_{k,k'},0) \cdot fr^{{up}^-}_{k'} \le F^+_k, \quad k \in K \end{aligned}$$

27 $$\begin{aligned} &(\phi ^{{up}^-}_k):~\sum _{n\in N} -PTDF_{n,k} \cdot ne_n \\&+ \sum _{k' \in K} \max (A_{k,k'},0)\cdot fr^{{up}^-}_{k'}\nonumber \\&+ \sum _{k' \in K} \max (-A_{k,k'},0) \cdot fr^{{up}^+}_{k'} \le F^-_k ,\quad k \in K \end{aligned}$$

28 $$\begin{aligned} &(\phi ^{{dn}^+}_k):~ \sum _{n\in N} PTDF_{n,k} \cdot ne_n \\&+ \sum _{k' \in K} \max (A_{k,k'},0) \cdot fr^{{dn}^+}_{k'}\nonumber \\&+\sum _{k' \in K} \max (-A_{k,k'},0) \cdot fr^{{dn}^-}_{k'} \le F^+_k, \quad k \in K \end{aligned}$$

29 $$\begin{aligned}& (\phi ^{{dn}^-}_k):~\sum _{n\in N} -PTDF_{n,k} \cdot ne_n \\&+ \sum _{k' \in K} \max (A_{k,k'},0) \cdot fr^{{dn}^-}_{k'}\nonumber \\&+ \sum _{k' \in K} \max (-A_{k,k'},0) \cdot fr^{{dn}^+}_{k'} \le F^-_k,\quad k \in K \end{aligned}$$

30 $$\begin{aligned} (\mu ^{up}_g):&~P^0_g + p_g + r^{up}_g \le Q^+_g, \quad g \in US \end{aligned}$$

31 $$\begin{aligned} (\mu ^{dn}_g):&~P^0_g + p_g - r^{dn}_g \ge Q^-_g, \quad g \in US \end{aligned}$$

32 $$\begin{aligned} (v^{up}_l):&~ dr^{up}_l \le QR_l, \quad l \in RL \end{aligned}$$

33 $$\begin{aligned} (v^{dn}_l):&~ dr^{dn}_l \le QR_l, \quad l \in RL' \end{aligned}$$

34 $$\begin{aligned}&r, dr, fr^{+}, fr^{-} \ge 0 \end{aligned}$$

Comparing the formulation to that of Section 2.1, the key difference is that our model will identify the maximum volume box within the feasible space of node-to-node reserve exchanges *nr*. These feasible exchanges are characterized by the DC network constraints, which are substituted by the auxiliary flow variables $fr_k$, based on Eqs. 24, 25. This substitution directly leads to the parameters $A_{k,k'} = PTDF_{FB_{k'},k} - PTDF_{TB_{k'},k}, ~\forall k, k' \in K.$ These parameters capture the impact of power injections on network elements, specifically when the effect is detrimental (i.e. when power flows in the direction of the constraint that we are trying to respect), without on the other hand reflecting alleviating effects (i.e. counter-flows on a line are ignored/eliminated). The objective function of the model is exactly the same as that of the "Caramanis" model. With Eqs. 26-29, it is guaranteed that reserves are matched such that the potential activation of reserves results in line loadings that respect line constraints. Eqs. 20-23 are nodal energy and reserve balance constraints, respectively. The remaining equations are similar to those of the model in Section 2.1. For a more detailed explanation of the model we refer the reader to [20]. In the present context, “cross-border trades” correspond to exchanges between distribution nodes.

## Analysis of Models

The two formulations that are described in section II are both fundamentally driven by a consideration of different scenarios for the loading of distribution lines. The key difference is that the inscribed boxes model is a *conservative approximation* of the polytope of feasible flows that corresponds to a complete enumeration of all possible activation scenarios. In this section we prove that, on a radial network with a single TSO demand at the transmission system, this approximation is *exact*.

### Equivalence Result

#### Proposition 1

The Caramanis and Inscribed-Boxes models are equivalent for radial networks.

#### Proof

The idea of establishing the equivalence of the two models is by providing mappings that move us from any feasible point in the space of the Caramanis model to a feasible point in the space of the inscribed-boxes model, and vice versa. Moreover, since the objective functions of the two models are identical in variables that are identical in both formulations, the objective function of the inscribed boxes model is less than or equal to that of the Caramanis model and vice versa, i.e. the two objective functions are equal. $\square$

#### Inscribed-boxes to Caramanis

**Fig. 1:**
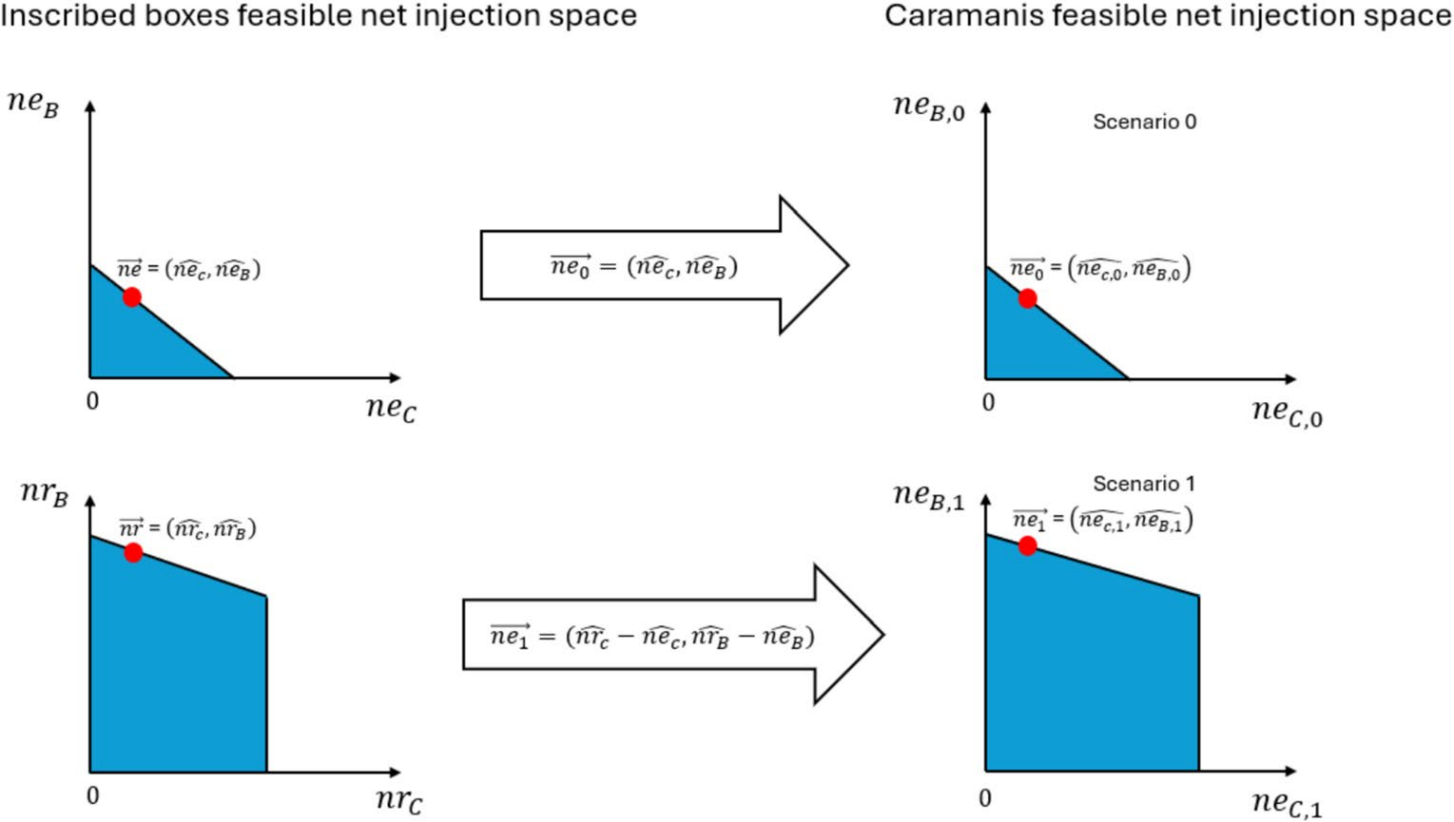
Mapping from the inscribed-boxes model to the Caramanis model in the feasible net injection space of a radial three-node example. Nodes B and C are exporting and node A is importing. Starting from the feasible net injection points $\vec {nr}$, $\vec {ne}$ of the inscribed-boxes model, Table 2 is used to obtain the corresponding feasible point $\vec {ne_1}$, $\vec {ne_0}$ of the Caramanis model

Table 2 provides the mapping that moves us from a feasible point of the inscribed-boxes model to a feasible point of the Caramanis model. In Appendix [23] we prove that if we start from a feasible point of the inscribed-boxes model (i.e. one that satisfies all constraints (20)-(34)) then the point in the left of Table 2 satisfies all constraints of problem (7)-(18). The direct mapping from a feasible point of inscribed-boxes to a feasible point of the Caramanis model is illustrated in the net injection space of a radial three-node example in Fig. 1.

**Table 2:** Inscribed-Boxes to Caramanis

#	Caramanis	Inscribed-Boxes
1	$dr^{up}_l$	$dr^{up}_l$
2	$dr^{dn}_l$	$dr^{dn}_l$
3	$p_g$	$p_g$
4	$r^{up}_g$	$r^{up}_g$
5	$r^{dn}_g$	$r^{dn}_g$
6	$pr^{up}_{g,1}$	$I^+_g(1) \cdot r^{up}_g$
7	$pr^{dn}_{g,2}$	$I^-_g(2) \cdot r^{dn}_g$
8	$ne_{0}$	*ne*
9	$ne_{1}$	$nr^{up} + ne$
10	$ne_{2}$	$-nr^{dn} + ne$
11	$f_{k,2}$	$$\sum \limits _{\begin{array}{c} k' \in K \end{array}} A_{k,k'} \cdot fr^{dn^+}_{k'} -\sum \limits _{\begin{array}{c} k' \in K \end{array}} A_{k,k'} \cdot fr^{dn^-}_{k'} + \sum \limits _{n \in N} PTDF_{n,k} \cdot ne_n$$
12	$f_{k,1}$	$$\sum \limits _{\begin{array}{c} k' \in K \end{array}} A_{k,k'} \cdot fr^{up^+}_{k'} - \sum \limits _{\begin{array}{c} k' \in K \end{array}} A_{k,k'} \cdot fr^{up^-}_{k'} + \sum \limits _{n \in N} PTDF_{n,k} \cdot ne_n$$
13	$f_{k,0}$	$\sum \limits _{n \in N} PTDF_{n,k} \cdot ne_n$
14	$\sum \limits _{l \in IS} imb_{l,1}$	$\sum \limits _{l \in RL} dr^{up}_l$
15	$\sum \limits _{l \in IS} imb_{l,2}$	$-\sum \limits _{l \in RL'} dr^{dn}_l$

#### Caramanis to Inscribed-boxes

**Fig. 2:**
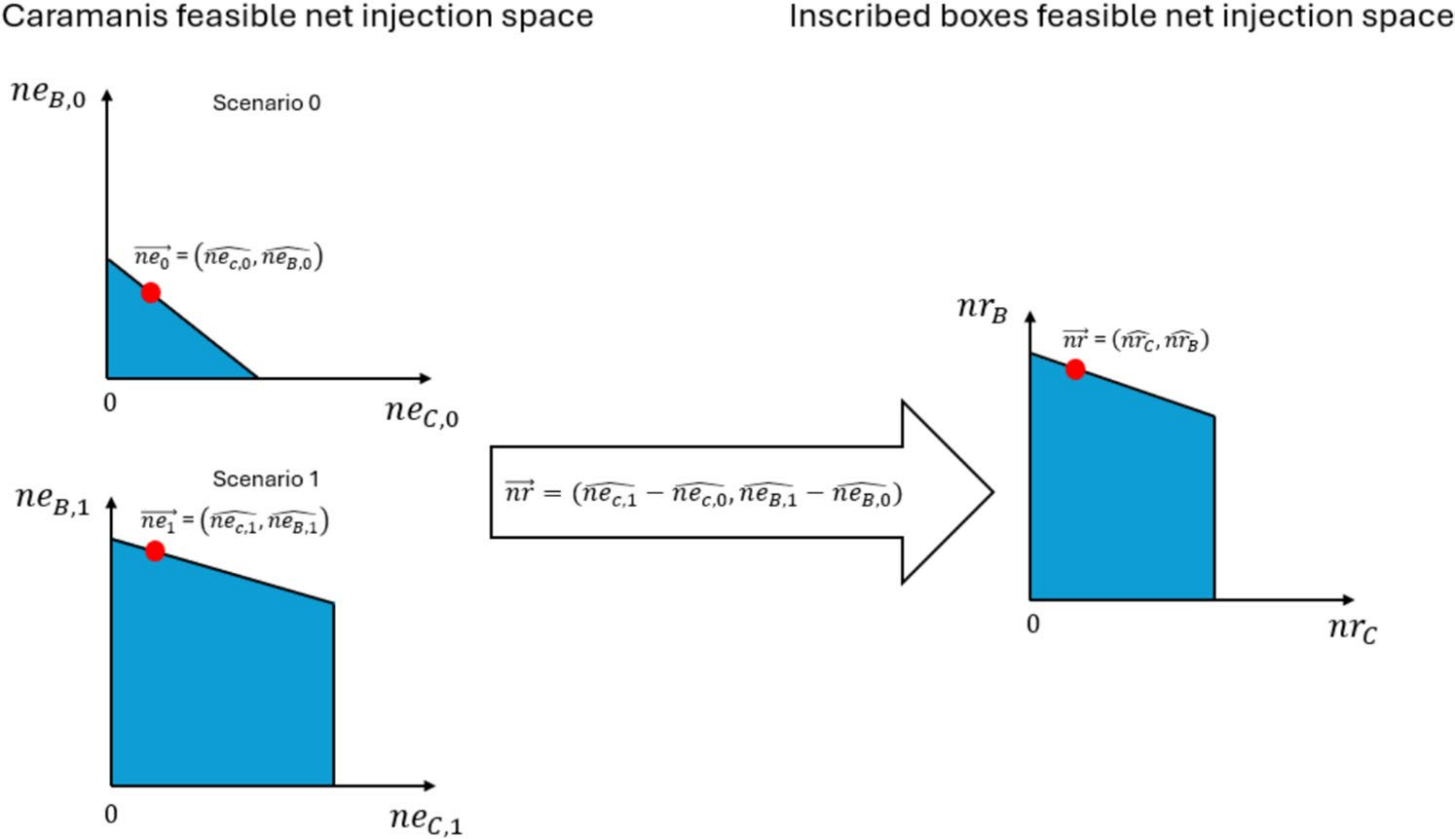
Mapping from the Caramanis model to inscribed-boxes model in the feasible net injection space of a radial three-node example. Nodes B and C are exporting and node A is importing. Starting from the feasible net injection points $\vec {ne_1}$, $\vec {ne_0}$ of the Caramanis model, Table 3 is used to obtain the corresponding feasible point $\vec {nr}$, $\vec {ne}$ of the inscribed-boxes model

We now move in the opposite direction. Table 3 maps a feasible point from the Caramanis model to a feasible point in the Inscribed-Boxes model. The detailed proof of why this is the case is provided in the Appendix [23]. The direct mapping from a feasible point of the Caramanis model to a feasible point of the inscribed-boxes model is illustrated, again in the net injection space of the same radial three-node example, in Fig. 2.

**Table 3:** Caramanis to Inscribed-Boxe

#	Inscribed-Boxes	Caramanis
1	$dr^{up}_l$	$dr^{up}_l$
2	$dr^{dn}_l$	$dr^{dn}_l$
3	$p_g$	$p_g$
4	$r^{up}_g$	$r^{up}_g$
5	$r^{dn}_g$	$r^{dn}_g$
6	*ne*	$ne_0$
7	$nr^{up}$	$ne_1 - ne_0$
8	$nr^{dn}$	$ne_2 - ne_0$
9	$fr^{up^+}_k$	$f_{k,1} - \sum \limits _{n \in N} PTDF_{n,k} \cdot ne_{n,0}$
10	$fr^{up^-}_k$	$-f_{k,1} + \sum \limits _{n \in N} PTDF_{n,k} \cdot ne_{n,0}$
11	$fr^{dn^+}_k$	$f_{k,2} - \sum \limits _{n \in N} PTDF_{n,k} \cdot ne_{n,0}$
12	$fr^{dn^-}_k$	$-f_{k,2} + \sum \limits _{n \in N} PTDF_{n,k} \cdot ne_{n,0}$
13	$\sum \limits _{l \in RL} dr^{up}_{l}$	$\sum \limits _{l \in IS} imb_{l,1}$
14	$-\sum \limits _{l \in RL'} dr^{dn}_{l}$	$\sum \limits _{l \in IS} imb_{l,2}$

### Duality Analysis

In this section we provide a duality analysis of our proposed inscribed-boxes model. This allows us to assign an economic interpretation to the dual variables of the model, and to understand the pattern of their behavior at the dual optimal solution.

Before proceeding with the detailed duality analysis, we provide some intuition about the optimal allocation of transmission capacity between energy and reserves, and connect this intuition to existing professional literature. Concretely, we refer the reader to [24]. Figure 1 of [24] presents the intuition that transmission capacity should be allocated at the level where the incremental increase in welfare due to the trade of reserve that is achieved by allocating additional transmission capacity to reserve becomes equal to the incremental decrease in welfare from the trade of energy which is a result of allocating limited transmission line capacity for the trade of reserve instead of the trade of energy. This point is clarified further with the following example and ensuing duality analysis.

**Fig. 3:**
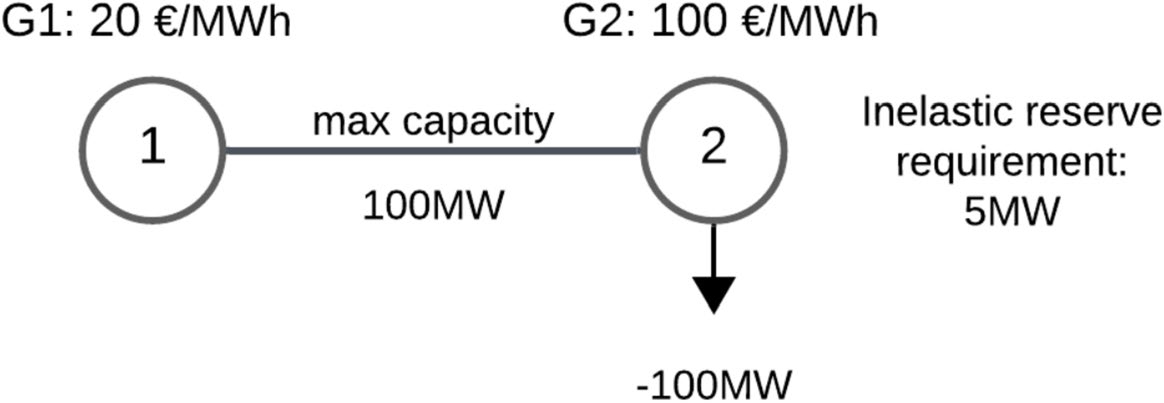
2-node network used for duality analysis of inscribed-boxes model

Consider the two-node system of Fig. 3. In this instance, we assume that a resource G1 can provide energy and reserve in node 1, a resource G2 can only provide energy at node 2, there is an inelastic demand of 5 MW of reserve in node 2 and there is finally an inelastic load of 100 MW at node 2. The capacity of the line that connects nodes 1 and 2 is 100 MW.

When we attempt to understand how the line capacity should be allocated optimally between the trade of reserve and the trade of energy, it is valuable to observe that the first increments of reserve are very valuable, because they enable the TSO at node 2 to cover its demand for reserve, and additional line capacity should be allocated up to the point where the incremental value from the trade of reserve equals the incremental welfare losses from not using this line capacity for the trade of energy.

The optimal allocation of the model is thus such that G1 covers the 5 MW of reserve demand, resulting in a reserve flow of $fr_{1-2}^+ = 5$ MW on the line. G1 also covers 95 MW of energy demand at node 2, resulting in an energy flow of $ne_1 = 95$ MW on the line.

Some of the dual variables of the inscribed boxes model admit a standard interpretation, which is based on the usual interpretations of energy and reserve co-optimization models. Concretely, $\lambda _n$ is the price of energy. $\lambda R_n^{up}$ is the price of upward reserve and $\lambda R_n^{dn}$ is the price of downward reserve. $\sigma$ is the negative of the price of energy at the hub node. $\mu _g^{up}$ is the profit margin of flexible assets in the upward reserve market. $\mu _g^{dn}$ is the profit margin of flexible assets in the downward reserve market. $v_l^{up}$ is the surplus of the TSO from the *l*-the increment of upward reserve capacity, while $v_l^{dn}$ is the surplus of the TSO from the *l*-the increment of downward reserve capacity.

Other dual variables admit a less standard interpretation, which is specific to our proposed formulation but where duality results uncover economically intuitive properties. We focus specifically on $$\kappa _n^{up}$$ and $$\kappa _n^{dn}$$, $$\phi _k^{up+}$$ and $$\phi _k^{up-}$$, $$\phi _k^{dn+}$$ and $$\phi _k^{dn-}$$. The first two are equal to the negative of the price of upward ($$\lambda R_n^{up}$$) and downward ($$\lambda R_n^{dn}$$) reserve, respectively, at node *n*. The rest of the dual variables are the marginal economical surplus of the TSO when trading energy and reserve (upward or downward) on a line *k*. Precisely, $$\phi _k^{{up}^+} = \lambda R_{TB_k}^{up} - \lambda R_{FB_k}^{up}$$$$= \lambda _{TB_k} - \lambda _{FB_k}$$ and $$\phi _k^{{up}^-} = \lambda R_{FB_k}^{up} - \lambda R_{TB_k}^{up}$$$$= \lambda _{FB_k} - \lambda _{TB_k}$$, while $$\phi _k^{{dn}^+}= \lambda R_{FB_k}^{dn} - \lambda R_{TB_k}^{dn}$$$$= \lambda _{TB_k} - \lambda _{FB_k}$$ and $$\phi _k^{{dn}^-} = \lambda R_{TB_k}^{dn} - \lambda R_{FB_k}^{dn}$$$$= \lambda _{FB_k} - \lambda _{TB_k}$$.

Given our interpretation of dual values, we note that the line capacity is indeed allocated in such a way that the incremental value of allocating to energy $\lambda _2 - \lambda _1 = 80$ €/MWh is equal to the incremental value of allocating to reserve $\lambda R_2^{up} - \lambda R_1^{up} = 80$ €/MWh. The proof of the above explanations is provided in the Appendix [23].

### Patterns of Energy and Reserve Prices

In this section we analyze the pattern of market clearing prices for different combinations of flow patterns. Again, without loss of generality in the case of radial networks, we focus on a two-node system. In the context of this section, we refer to upstream and downstream with respect to the transmission network and we assume that ORDC is always at the transmission level.

The flow patterns that we consider are the following, taking into account that TSO demand in our TSO-DSO coordination model always appears at the high-voltage transmission system:

- Flow pattern 1: energy flows upstream, upward reserve flows upstream
- Flow pattern 2: energy flows downstream, upward reserve flows upstream
- Flow pattern 3: energy flows upstream, downward reserve flows downstream
- Flow pattern 4: energy flows downstream, downward reserve flows downstream

**Fig. 4:**
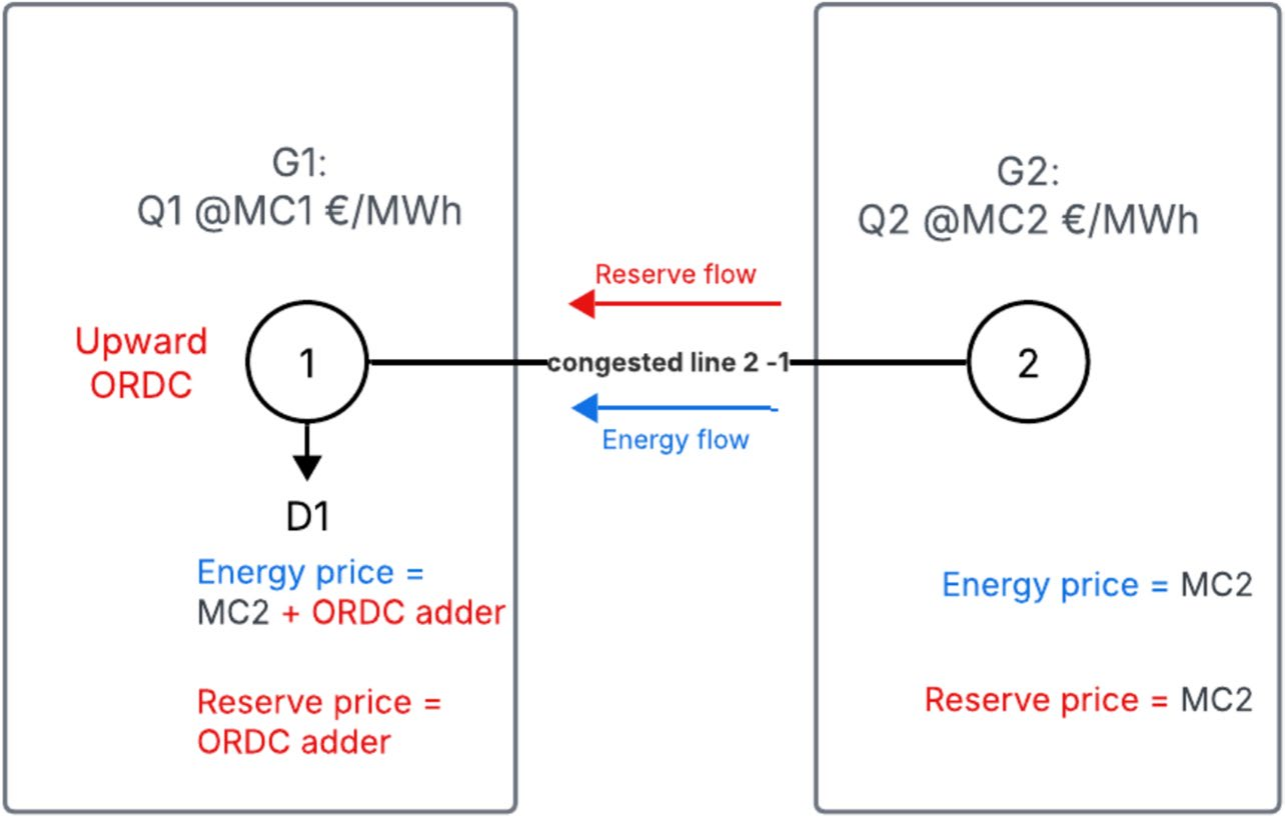
Network for flow pattern 1, where there is a negative imbalance at the transmission network and the reserve demand is also located at the transmission network. In this case, $MC_2 < MC_1$

We provide a duality analysis which allows us to extract a price pattern in the Appendix [23]. In what follows we describe the price patterns and their drivers. The analysis can be generalized to the case of non-congested subnetworks connected to each of the two nodes. *Flow pattern 1 - Energy flows from distribution to transmission network, with upstream congestion, upward reserve demand at transmission network*. In Fig. 4 depicts this flow pattern using a simplified illustrative example. The reserve price at the transmission level is equal to the ORDC valuation due to scarcity conditions. In addition, the flexible resources at the distribution level are not used up to their capacity, thus their scarcity rent in both energy and reserves are equal to zero. This implies that the energy price at the distribution level equals the marginal cost of the marginal unit at the distribution level, and the reserve price at the distribution level equals zero. Given the property established in section III-B whereby the difference in energy prices along the nodes of the congested line equals the difference in reserve prices along those same nodes, we conclude that the energy price at the transmission level equals the energy price at the distribution level plus the ORDC adder. *Flow pattern 2 - Energy flows from transmission to distribution network, with upstream congestion, upward reserve demand.* In this flow pattern, the reserve price at the transmission level is equal to the ORDC valuation due to scarcity conditions. The generator at the transmission level is the marginal unit and splits between markets. At the distribution level the generator provides upward reserve causing a congestion on the line and thus resulting in a reserve price valued at zero and an energy price equal to the marginal cost of the marginal generator. Again, the difference of energy and reserve price along the nodes should be equal, meaning the energy price at the transmission level is the energy price at the distribution level plus the ORDC adder. *Flow pattern 3 - Energy flows from distribution to transmission network, with downstream congestion*^3^, *reserve demand downward*. In this pattern, the downward reserve price at the transmission level is equal to the ORDC valuation due to scarcity conditions. The flexible resources at the distribution level are not used up to their capacities, resulting in zero profit margins in both the energy and reserve markets. This implies a reserve price of zero at distribution level, and an energy price equal to the marginal cost of the distribution level resource. This in turn implies that the energy price at the transmission system is equal to the energy price of the distribution system *minus* the transmission system reserve price. This stems from the equilibrium conditions of the network operator: the value lost by upstream energy flow should equal the value generated by downstream reserve flow. Note that energy flows from the more expensive to the cheaper location in this setting, which is contrary to the intuition of energy-only models.

*Flow pattern 4 - Energy flows from transmission to distribution network, with downstream congestion, downward reserve demand.* The same applies as for pattern 3, i.e. G2 splits its capacity between the energy and reserve markets, which in turn implies a zero price for reserve at the distribution level and an energy price equal to its marginal cost. The difference is that less downward reserve is provided, as the energy flows at the same direction as the reserve. The reserve price at the transmission level is determined by the partially served ORDC. And the no-arbitrage conditions of the network operator finally dictate that the energy price equals the energy price at the distribution level minus the reserve price.

It can be concluded that the energy price, when congestion occurs, equals the marginal cost of the marginal unit plus or minus the ORDC adder for nodes at the source (from node) of the congested line, whereas for nodes at the destination (to node) of the congestion the energy price equals to the marginal cost of the marginal unit, meaning also that the location of the marginal unit is irrelevant. The overall pattern is depicted in Table 4.

It is worth noting that the price patterns discussed above occur when the marginal unit splits its capacity between energy and reserve. More complicated patterns can occur in the presence of additional constraints, e.g. ramps.

**Table 4:** Pattern of energy and reserve prices along congested lines of a radial network in various scenarios of energy and reserve flows

	Energy flow	Upward reserve flow	Downward reserve flow	Energy price upstream	Energy price downstream
Pattern 1	Downstream to upstream	Downstream to upstream	N/A	MC + ORDC adder	MC
Pattern 2	Upstream to downstream	Downstream to upstream	N/A	MC + ORDC adder	MC
Pattern 3	Downstream to upstream	N/A	Upstream to downstream	MC - ORDC adder	MC
Pattern 4	Upstream to downstream	N/A	Upstream to downstream	MC - ORDC adder	MC

## Case Study

In this section we present a case study of transmission-distribution coordination in Belgium. In section IV-A we describe our methodology for setting up the case study, and we compare the alternative designs and their effect on the profitability potential investments in section IV-B.

### Setup of Case Study

#### Day types.

We model a year of operation using 8 representative day types, 1 weekday and 1 weekend day per season. The load, renewable supply and import profiles vary by day type.

#### Transmission network.

The Belgian transmission network is an updated version of [25]. It consists of 30 380-kV buses, 30 lines.

#### Renewable supply and loads.

Loads and renewable generation data are sourced from the ENTSO-E transparency platform. They are modeled as price-inelastic power withdrawals and injections respectively, and are thus not flexible in the real-time market.

#### High-voltage power units.

There are 39 generators in the system. We use a unit commitment model based on [26] in order to determine the commitment schedule of power generation units in Belgium for every hour of the day for each studied day type. This unit commitment model also indicates the amount of imports at each node of the Belgian system. Pumped hydropower is assumed to offer a fixed amount of reserve at every time step (which is determined in the unit commitment model) whereas run of river hydropower is considered as price-inelastic withdrawal or injection and is thus directly incorporated in the net demand data of the model. The ramp limits of units are adjusted in order to be consistent with the full activation time of the reserve that we analyze in our case study, in particular a full activation time of 7.5 minutes is assumed.

#### Imports.

The unit commitment model that we use is based on a zonal representation of the network. In order to disaggregate the zonal information of our source model to nodal information required for the analysis conducted in the present paper, we use the flow-based constraints of the zonal model in order to identify flows between Belgium and neighboring zones (after rescaling the flows on these flow-based constraints so that the net position of Belgium becomes consistent with these flows). Subsequently, these cross-zonal flows are disaggregated to individual physical lines in proportion to the physical capacity of each line.

#### Real-world distribution network (DN1).

The first distribution network that we use relies on a commercial dataset. The network consists of 235 buses and 247 edges, and is thus non-radial. In order to derive a radial network from this dataset, we compute the maximum spanning tree of the original network, which consists of 235 buses and 234 edges. The capacity of four transformers and certain lines was scaled up in order to render the AC optimal power flow on the radial system feasible.

#### Xanthi distribution network (DN2)

[27]. The network consists of 99 buses and 98 edges. The generators in the dataset include minimum/maximum limits. The dataset includes fixed injections and offtakes of real and reactive power at each bus, we only retain the real power data in our analysis.

#### Interface with distribution networks.

In our analysis, we replace certain nodes in the transmission network of Belgium by detailed distribution networks. The day-ahead consumption of the distribution networks is locational but static (i.e. spatially but not temporally differentiated). In order to introduce temporal variability, we scale the spatial consumption of each distribution node according to the transmission system time series. Distribution system load is assumed to be a price-inelastic fixed injection in the network. Flexible resources are instead assumed to be active in the real-time market, which is the focus of our analysis.

The final Belgian network that is used in our study includes 6 distribution networks of type DN1 and 1 distrbution network of type DN2. The distribution networks are placed in transmission buses that correspond to major Belgian cities. The load profiles and flow limits of the distribution networks are scaled according to the initial load of the corresponding transmission network bus. The renewable generation corresponding to the replaced transmission bus is retained. The overall network consists of 1503 distribution nodes, 1533 nodes in total and 1533 edges.

The marginal costs of the transmission network flexible assets are provided in [26], whereas the marginal costs of the assets that are located in the distribution network are based on empirical data that were derived from the FEVER EU Horizon 2020 project [28].

The market models are implemented in Julia v.1.9.4 using JuMP v1.21.1, on an Asus Zenbook 14 with a 1.80GHz Intel Core i7-8550U processor with Windows 10 (64-bit). The chosen linear programming solver is Gurobi 11.

#### ORDCs.

We focus on a single reserve product in the case study. Specifically, we analyze upward reserve with a full activation time of 7.5 minutes. ORDCs vary by season and 4-hour interval of the day. The ORDCs are sourced from the work of [29], where the authors have precisely calibrated ORDCs for the Belgian system.

#### Imbalances.

Imbalances at the slack bus of the transmission network are sampled independently as random values from a normal distribution $\mathcal {N}(0 \text { MW}, 100 \text { MW})$ for each time step.

### Comparison of Different Designs

**Fig. 5:**
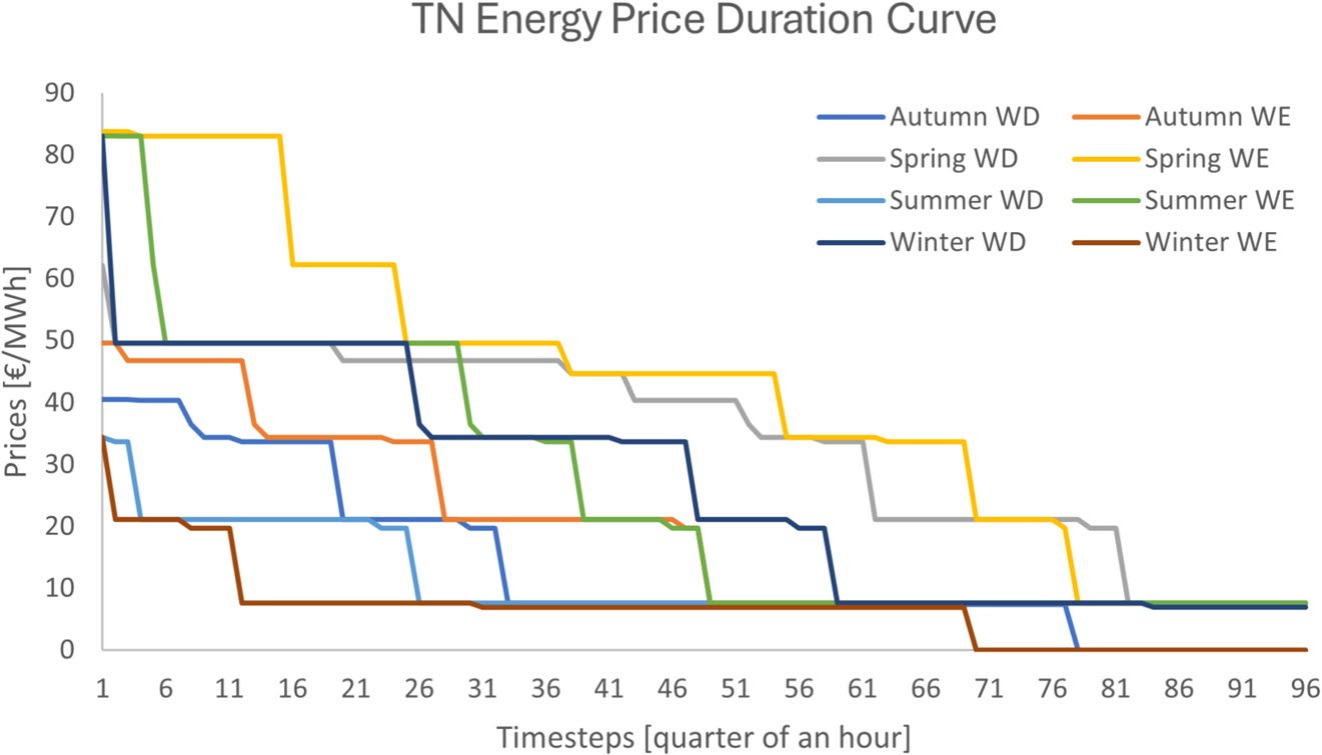
Transmission system energy price duration curve for designs A and B

**Fig. 6:**
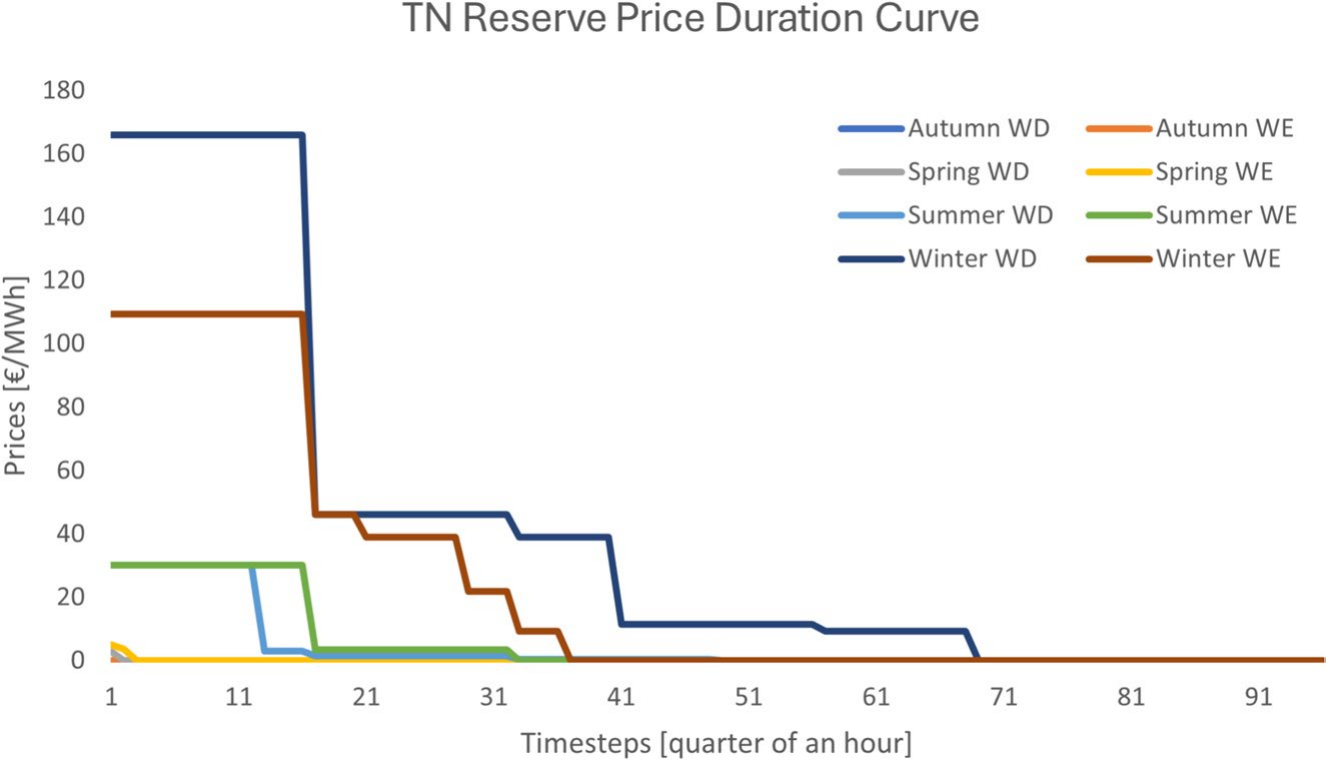
Reserve price duration curve for design B

**Fig. 7:**
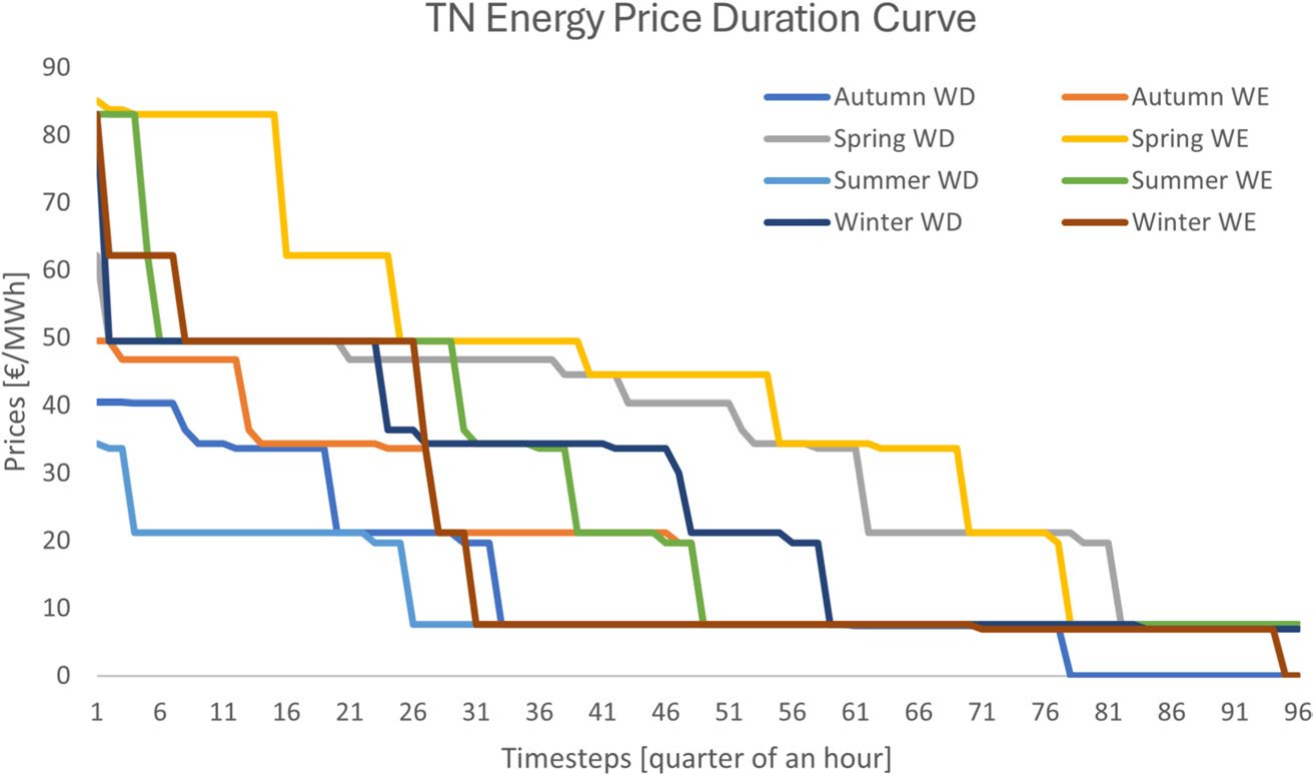
Transmission system energy price duration curve for design C

**Fig. 8:**
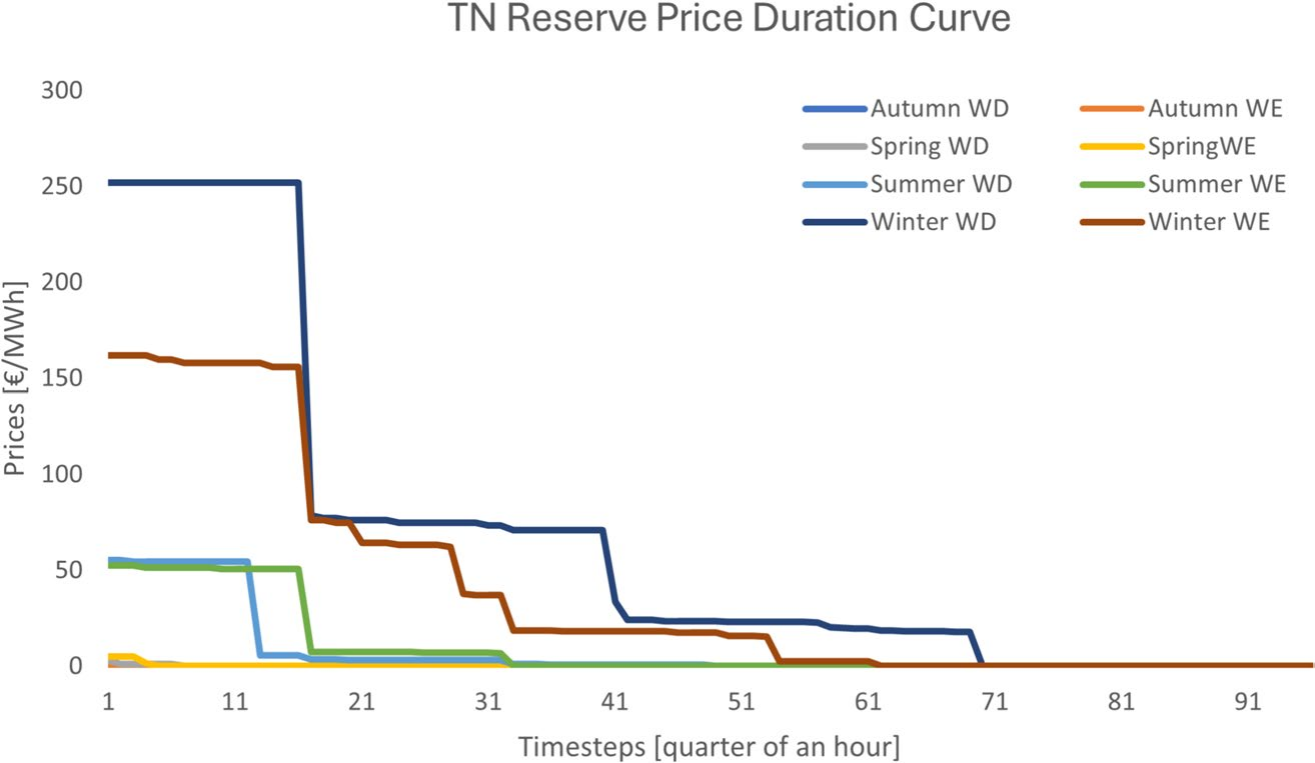
Reserve price duration curve for design C

In this section we analyze the effect of alternative designs on the investment incentives of a candidate flexible asset. We specifically focus on a natural gas asset with an amortized investment cost of 5 €/MWh and a marginal cost of $MC=80$ €/MWh. The investment cost corresponds to an overnight cost of 400 €/kW, a lifetime of 25 years, an interest rate of 12%, and annual compounding.

For a unit of considered capacity, $P^+=1 MW$, the profit maximization problem of a flexible asset owner who faces an energy price $\lambda _t$ and a reserve price of $\lambda R_t$ is at time step *t* is:

$$\begin{aligned}&\underset{p,r \ge 0}{\text {max}}\quad \lambda R_t \cdot r + (\lambda _{t}- MC)\cdot p \\&r \le R \\&p + r \le P^+ \\ \end{aligned}$$

We consider two alternative levels of flexibility in our case study, namely ramp limits at 10% and 75% of nameplate capacity, i.e. $R=0.1$ and $R=0.75$.

Alternative market designs result in alternative revenue streams in the energy and reserve markets, i.e. different time series $\lambda _t$ and $\lambda R_t$. We consider the following alternative designs:

- Design A: reserve deliverability is not considered and there is no scarcity pricing
- Design B: reserve deliverability is not considered and there is scarcity pricing
- Design C: reserve deliverability and scarcity pricing are both considered.

We then analyze the incentives of installing the facility at different areas of the network. Specifically, we consider the possibility of installing the capacity at the transmission network, a distribution network without congestion (typical of DN1) and a distribution network with congestion (typical of DN2).

*Design A*. The pricing results of the case study under design A are depicted in Fig.5. The only revenue stream is from the energy market and the average energy price at the transmission system over the year is 21.96 €/MWh.

*Design B*. The pricing results of the case study under design B are depicted in Figs. 5 and 6. This design collects revenue streams from both the energy and reserve markets due to the introduction of scarcity pricing. The average energy price at the transmission system over the year is 21.96 €/MWh, whereas the average reserve price is 10.35 €/MWh. It is worth mentioning that Autumn does not exhibit reserve scarcity, whereas most of the scarcity is observed during the winter.

*Design C*. The pricing results of the case study under design C are depicted in Figs. 7 and 8. As in the case of design B, this design also generates revenue streams from both the energy and reserve markets due to the introduction of scarcity pricing. The average energy price at the transmission system over the year is 22.94 €/MWh, whereas the average reserve price amounts to 17.04 €/MWh. The increase in reserve prices results from the congestion that is caused by reserve flows. In particular, less reserve is delivered to the TSO, since distribution network flexible assets cannot deliver reserve to the transmission system due to congestion at the distribution system.

*Profit results for assets in transmission network*. The profitability of investments under different configurations is summarized in Table 5, bold cells indicate combinations of types of assets and designs whose profits are below the threshold of investment cost (5 €/MWh), and thus fail to be profitable. When the asset under consideration is inflexible (10% ramp) the investment is not viable under any design. For design A, the profit is below the investment cost for each type of generator. In contrast to design B, when deliverability is considered (design C), profits in the transmission network increase due to an increase in reserve prices caused by the fact that less reserve is delivered due to congestion at the distribution level.

*Profit results for assets in distribution network DN2*. DN2 is representative of a distribution network without congestion. The absence of congestion in the distribution network implies that the distribution prices become equal to those of the transmission network. Thus, the same outcomes prevail as in the case of transmission network assets.

*Profit results for assets in distribution network DN1*. In the case of an inflexible asset (10% ramp) investment is worthwhile (with a small margin) in an area with local congestion, in contrast to the other two areas. For the highly flexible asset (75% ramp), design C decreases the profitability of the flexible asset close to the investment cost in contrast to design B, due to the fact that reserve of the flexible asset cannot be delivered to the transmission network. Thus, considering reserve deliverability can discourage an investment in assets that are placed behind a congestion. Design B exhibits greater profitability for an asset within DN1 relative to the alternative locations. This increased profit potential behind congested lines is mainly due to the higher prices that typically result from the activation of a more expensive generator.

## Conclusions

**Table 5:** Profitability of different types of assets under different designs, in €/MWh

	Asset	TN	DN1	DN2
Design A	Flexible	**0.0504**	5.835	**0.0504**
	Inflexible	**0.0504**	5.835	**0.0504**
Design B	Flexible	7.819	13.604	7.819
	Inflexible	**1.086**	6.871	**1.086**
Design C	Flexible	12.828	5.830	12.828
	Inflexible	**1.757**	5.830	**1.757**

We propose a computationally tractable model for representing reserve deliverability using a formulation based on inscribing boxes in polyhedra, and generalize the formulation for a meshed network. The equivalence of the inscribed boxes formulation to an alternative deliverability formulation based on work by Caramanis[18] is proven for radial distribution networks. The economic dimension of the dual variables and the properties of the DLMPs resulting from our proposed model are analyzed. We conduct a realistic case study of transmission-distribution system coordination in Belgium. Our interest is on understanding the extent to which the model can stimulate through scarcity pricing and reserve deliverability on contemplated investments in flexible assets. The study indicates that scarcity pricing alone can exaggerate the profitability of flexibility investments, while the inclusion of reserve deliverability provides a more realistic reflection of network limitations. In particular, the analysis highlights that assets located behind distribution network congestion experience diminished investment incentives when deliverability constraints are considered, thus emphasizing the need for network-aware market mechanisms. The introduction of deliverability constraints increases reserve prices at the transmission level, improving the revenue potential of assets in uncongested locations and ensuring that price signals reflect scarcities more accurately.

The discussion of future research directions builds on these findings. Future work should focus on developing decomposition methodologies for the proposed optimization problem to enhance scalability and enable applications to systems of arbitrary size, integrating AC network flows to assess the feasibility of market outcomes, and extending the formulation to a less conservative approach for non-radial networks.

## Data Availability

The paper provides access to public datasets via hyperlinks. One specific dataset mentioned in the paper cannot be made publicly available due to its commercial nature.

## Footnotes

1 Reserve deliverability refers to the practice of committing reserves in a way that accounts for the ability of the network to deliver the committed reserves at different parts of the system [18, 19].

2 We also refer to agents that can provide reserve as flexibility service providers (FSPs) throughout the text.

3 Note that since we assume scarcity of downward reserve at the transmission system, there is no interest in considering upstream congestion.
